# Determinants of Vietnamese footwear exporting firms’ market selection: A multinomial logistic analysis of panel data

**DOI:** 10.1016/j.heliyon.2019.e02582

**Published:** 2019-10-18

**Authors:** Quan-Hoang Vuong, Thi-Hanh Vu, Quang-Hung Doan, Manh-Tung Ho

**Affiliations:** a Centre for Interdisciplinary Social Research, Phenikaa University, Yen Nghia, Ha Dong, Hanoi 100803, Viet Nam; b Faculty of Economics and Finance, Phenikaa University, Yen Nghia, Ha Dong, Hanoi 100803, Viet Nam; c School of Economics and International Business, Foreign Trade University, Hanoi, Viet Nam; d Faculty of Basic Science, Foreign Trade University, Hanoi, Viet Nam; e Centre for Interdisciplinary Social Research, Phenikaa University, Hanoi, Viet Nam

**Keywords:** Economics, Vietnam, Export firms, Direction of trade, Multinomial logit, Footwear exports, The United States, The European Union

## Abstract

The literature on export activities is dominated by studies on the determinants of export performance, we contribute to this literature by investigating the determinants of selection of markets by using panel data of Vietnam's footwear firms for the 2006–2010 period. Since no variance was found between firms, a pooled multinomial logit model is consequently preferable. Among the notable results, export value shows a positive correlation with footwear firms serving the US and EU markets. Although Vietnamese footwear firms are less likely to export to the ASEAN countries, they tend to focus on diversifying their products in this market. Both private and FDI firms are less likely to export to the EU compared with their state-owned counterparts (SOEs). However, private firms outperform SOEs in the U.S market.

## 1 Introduction

Around the world, recent economic insecurities have brought about the rise of populist politicians and with it, the turn against globalization. Global events such as an inconclusive Brexit deal and the ongoing trade war between the U.S. and China are hurting investors' confidence and posing a great threat to the global economy. It is estimated that international trade as a share of Gross Domestic Products (GDP) fell from 60% in 2011 to 56% in 2016 (Kopf, 2018). Against this global trend, Vietnam's trade as a percentage of GDP increased more than four times in 2017.

Since the country implemented its economic reform—*Doi Moi* policy in 1986, the gradual economic liberalization and internationalization have yielded an impressive outcome: more than 30 consecutive years of growth, which has brought Vietnam's GDP per capita from US$421 in 1986 to more than US$2,000 in 2015 (Pham and Vuong, 2009; Vuong, 2014; Vuong et al., 2019). In this success story, international trade has played a central role. Vietnam has signed 12 free trade agreements involving 25 countries such as China, Japan, Korea, Russia, ASEAN countries, to name a few, and currently it is negotiating new trade deals with 33 other countries including 28 EU countries (WTO Center, 2019).

The export value of Vietnam increased remarkably from US$39.8 billion in 2006 to US$132 billion in 2013, equivalent to 60% and 77.1% of total GDP respectively. Notably, the Vietnamese footwear industry ranks third in export value after crude oil and textiles, making up for about 7.2% of the total export turnover of Vietnam from the period of 2006–2013 (General Statistics Office of Vietnam, 2013). Given the overall importance of the footwear industry to Vietnamese import-export, and the strategically critical role of the destination market selection, this paper sets out to examine factors such as ownership, age, firm performance, number of products or total export value that associate with the choice of a market of Vietnamese footwear exporting firms. Next, the current literature on the issue, which largely focus on exporting firms’ performance, will be briefly examined.

Firms in their process of internationalization may face both external and internal challenges. In the internationalization process, firms tend to expand their scope of activities to increase their economies of scale. As a result, there is a causal relationship between economies of scale and international trade; countries with the relatively large share in the domestic market are more likely to be an exporter of such goods (Krugman, 1980). Sleptsova (2011) explained that economies of scale exhibited a variation of positive and negative effects on different sectors when it comes to exporting from Ukraine to the EU. With firms coming from small domestic markets, the effect of economies of scale is very diminutive in determining the performance of these firms in foreign markets (Ethier, 1979; Helpman, 1984).

Additionally, scholars in international trade have studied product diversification. Hotopp et al. (2005) examined how exporters' performance was associated with by-product specialization or diversification and showed that firms being able to develop more diverse products had better export sales than those are not. Arrow (1972) postulated a theory of learning by doing whereby firms can learn by exporting an increasing number of new products. In this case, launching a new product presents a firm's innovation capacity. In contrast, Baldwin and Gu (2004) emphasized on product specialization which implied that when entering export market, firms tend to focus on a particular range of products rather than a variety of items which allows for exploitation of scale economies. Similarly, Amable (2000), Laursen (2000) and Peneder (2003) demonstrated the impact of product specialization on export trade through their empirical results. However, Funke and Ruhwedel (2001) found that product diversification is only significant in the industry of capital-intensive products, while in that of labor-intensive, more diverse products do not express any inconsistency in export performance.

For a long time, at firm-level internationalization, business governance has been taken as the main determinant for the successful establishment of export firms. In recent theories about international trade, the focal point has been shifted to another entity, either a firm's productivity or the firm's efficiency. This notion has broadened the scope of research of trade internationalization at micro-level. Melitz (2003) featured the firm's productivity as the major determinant for export firms in entering foreign markets. Furthermore, studies in different parts of the world such as Colombia, Mexico, Morocco, the US, or Taiwan have found a similar pattern: productive firms tend to be more adaptable to confront the adversity of foreign markets than other ones (Aw et al., 2000; Bernard and Jensen, 1999; Clerides et al., 1998). Moreover, Sleptsova (2011) and Bernard and Jensen (2004) went further to affirm the prerequisite role of productivity in determining firms' success when entering foreign markets. Meanwhile, Wagner (2007), in a meta-analysis of 54 micro-econometric studies from 34 countries, stressed that there is no such strong correlation between export activity and labor productivity or total factor productivity. Likewise, the question of whether exporting, in turn, increased productivity is also raised by Van Biesebroeck (2005), De Loecker (2007), Mukim (2011), Delgado et al. (2002).

Age of a firm can also be an important factor in understanding firms’ choices of markets. Older firms can accumulate skills, as such, they are expected to perform better than the younger (Fakih and Ghazalian, 2013; Iyer, 2010; Javalgi et al., 2000; Majumdar, 1997). However, one can also argue that older firms can be less flexible to adapt to new markets, resulting in lower export performance compared with the younger one (Amornkitvikai et al., 2013).

Type of firm ownership also plays an important role in firm performance; one would expect the difference in terms of organizational characteristics and managerial styles could lead to different performance outcomes. Many studies found that state-owned firms perform worse than foreign firms (Aggrey et al., 2010; Farole and Winkler, 2011; Javalgi et al., 2000; Özçelik and Taymaz, 2004; Rankin et al., 2006).

The literature on export activities tends to deal with the determinants of exporting firm's performance rather than the determinants of exporting firms' choice of market destination (Baliamoune-Lutz, 2019; Boehe et al., 2016; Fugazza and McLaren, 2014; Tinashe Kahiya and Dean, 2014). Therefore, our study will attempt to address this gap in the literature by answering the following research questions:

**RQ1:** Is there any systematic difference between Vietnamese footwear firms exporting to the US, EU and ASEAN markets?

**RQ2:** Does the export scale affect the destination markets of footwear firms such as the US, EU, and ASEAN markets? In other words, do economies of scale stimulate footwear firms’ exports to these markets?

**RQ3:** Does export diversification relate to the market selection of Vietnam's footwear firms?

**RQ4:** Do the FDI footwear firms tend to be more active in more distant markets such as the EU and the US compared with the private and State-owned enterprises (SOEs)?

**RQ5:** Do the age and productivity of a footwear firm matter given that footwear firms want to diversify export markets?

The remainder of this paper is organized as follows. Section 2 explains the methodology and data. Based on the data, Section 3 gives an overview of Vietnam's footwear exports for a 2006–2010 period to provide the most comprehensive background for the research. The regression results follow in section 4. The last section discusses the results and concludes the paper.

## 2 Materials and methods

### 2.1 The model

We apply the multinomial logit model to measure and analyze the determinants affecting the choices of market entry of Vietnamese footwear firms including the US, EU and ASEAN markets. This model allows us to identify the percentage of firms exporting to any markets in a particular year and the maximum value of exports gained by a firm as follows:

(1) $$P_{in}=\frac{\text{exp}\left(V_{in}\right)}{\exp\left(V_{im}\right)+\exp\left(V_{in}\right)+\exp\left(V_{iq}\right)+\text{exp}\left(V_{ip}\right)}$$

• Where $V_{in}$is the utility function of the destination country *n* for firm *i*
• $V_{im}$is the utility function of destination country *m* for firm *i*
• $V_{iq}$is the utility function of destination country *q* for firm *i*
• $V_{ip}$ is the utility function of destination country *p* for firm *i*
• $V_{in}=\alpha +\beta_{1}X_{1}+\beta_{2}X_{2}+\ldots +\beta_{k}X_{k}$
• And $P_{in}$is the probability of market entry *n* of firm *i*

The multinomial logit which is applied in this study includes:

* Dependent variable: includes four nominal variables such as the US, EU, ASEAN and NEAU (the country group does not cover EU, US and ASEAN countries).

* *Independent variables*:

• The total value of export of firm *i* in year *t* measures a firm's export value.
• The number of footwear products of firm *i* in year *t* measures the product diversification of a firm.
• Labor productivity of firm *i* in year *t* is measured by dividing a firm's revenue by its total number of employees.
• Age of firm is identified upon the year of establishment.
• Dummy variable SOE takes the value 1 if a firm is owned by the state or zero otherwise; Private variable takes the value 1 if a firm is owned by a single individual or zero otherwise; FDI variable takes the value 1 if its state is owned by a person or company from a foreign country or zero otherwise.

### 2.2 Data

This paper uses microdata of Vietnam's footwear firms for a period from 2006 to 2010. The dataset includes firm identity code, the name, and code of the importing country, transaction code, currency code, exchange rate, export volume, unit price, and export value in the foreign currency, name of products and their code at 10-digit SITC level. The data were supplied by the General Department of Vietnam's Customs (GDVC). This government body is responsible for managing export and import activities of firms in Vietnam as well as collecting data on their trading activities. Firms that need to export or import goods are required to complete a declaration sheet and submit to a local branch at a border gate of the GDVC. In details, the first dataset contains 128 footwear firms involving in export activities. There are 15 different currencies used for trade transactions, the transaction value in foreign currencies is converted to Vietnamese Dong (VND) using the exchange rate notified by Vietnam's State bank at the date of the transaction.

## 3 Background

During the 2006–2010 period, the dataset recorded that 128 enterprises were involved in the export of footwear products. These firms have been able to export to many countries around the world, especially to the prominent economies such as the EU, US, ASEAN, China, and Japan. According to the data supplied by the GDVC, from 2006 to 2010, the US was the biggest partner of Vietnam's footwear, recording nearly US$1.5 billion transaction in 2010. It was followed by EU countries with US$ 2.5 billion in the same year. Table 1 presents the percentage of firms by the number of markets, to which each firm can export. As can be seen, the majority of Vietnamese footwear exporting firms have been able to diversify their market destinations with nearly 55% of firms penetrating more than two markets.

**Table 1:** Percentage of footwear firms by the number of markets in the 2006–2010 period.

Number of export markets	Period 2006–2010	
	Percentage of firms (%)	Percentage of export value (%)
1	45.69	0.41
2	8.73	0.7
3	5.97	0.39
4	3.1	0.23
5	2.66	0.54
6	2.09	0.75
7	1.69	0.57
8	1.82	0.36
9	1.02	0.37
10	0.87	0.94
>10	1.06	94.5
Number of markets per firm	9.17	
Maximum number of markets per firm	88	
Number of firms	128	

*Note: Figures are calculated by the authors, using STATA 14.0.

It is also noteworthy that although 45% of firms export to only one market, their export value was very low, capturing only 0.41% of the total export value. Conversely, only 1.05% of firms can export to more than ten markets, but they account for most of the revenue, recording 94.5% of total export value. It shows that Vietnamese footwear firms are very different in terms of export capacity.

**Fig. 1:**
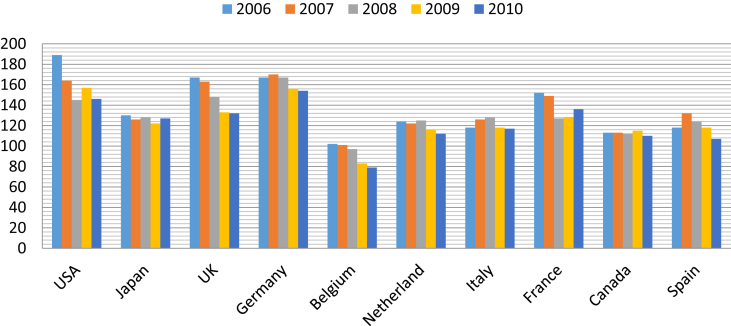
The total number of Vietnam's footwear firms to some destination markets from 2006-2010 (Unit: number of firms; Source: General Department of Vietnam's Custom).

**Fig. 2:**
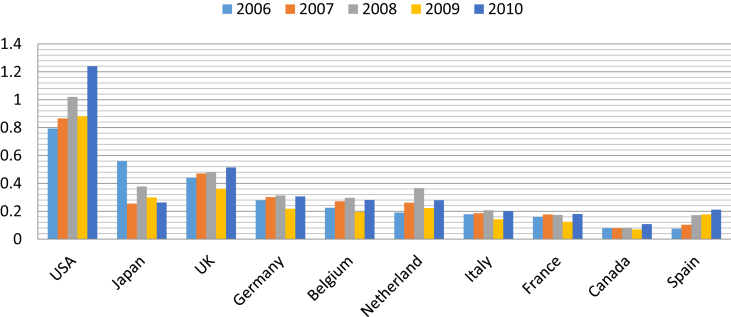
The export values of Vietnam's footwear firms to some destination markets from 2006-2010 (Unit: billion USD, Source: General Department of Vietnam's Custom).

The charts below show the total number as well as the export values of Vietnam's footwear firms to top ten prominent markets from 2006 to 2010. Fig. 1 clearly shows that countries with strong economic power such as the US, UK, and Germany are the most attractive destination markets to Vietnamese firms. In 2006 alone, there were nearly 190 Vietnam's footwear firms exporting to the US. Notably, the number of Vietnam's footwear firms exporting to these countries has been decreasing with time, yet the export values have been on the upward trend, as seen in Fig. 2. There are less and less Vietnam's companies exporting to the US, but the value that they brought back has been growing over time and registered as the one with largest export values of approximately US$1.2 billion in 2010.

Japan as a prominent market of Vietnam's footwear products has been noted with the reduction in value throughout years from over US$0.55 billion in 2006 to less than US$0.3 billion in 2010. Meanwhile, although the number of firms exporting to the US, UK, and Germany is comparable to each other, the export values to UK and Germany are less than a half of that to the US. This phenomenon indicates the fact that most of Vietnam's footwear exporting firms to the US can conclude high valued contracts. By comparison, firms which export to other countries such as France, Spain, Canada, and Italy are almost small and medium-size ones.

**Fig. 3:**
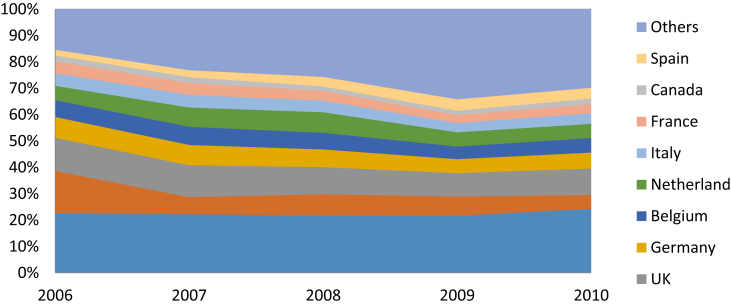
Footwear export value ten largest destination markets from 2006-2010 (Unit: %; Source: General Department of Vietnam's Customs).

Fig. 3 illustrates the proportions of the export value of Vietnam's footwear exports firms broken by destination markets. Overall, the percentages of export values to the US, UK, Germany, and Belgium have been steady throughout the years. The total exports to the US account for a large amount at over 20% over the period. As seen from previous descriptions, the export value to Japan has been declining throughout the years which were seen in the reduction in the percentages over the total export value from 16% in 2006 to 5.2% in 2010.

For country groups, Vietnam's footwear products were exported to 24 countries in EU region including Austria, Belgium, Bulgaria, Cyprus, Germany, Denmark, Spain, Estonia, Finland, France, Greece, Hungary, Italy, Lithuania, Luxembourg, Latvia, Malta, Netherlands, Poland, Portugal, Slovakia, Slovenia, Sweden and Ukraine. Similarly, the ASEAN group consists of Thailand, Brunei, Laos, Malaysia, Singapore, Philippines, Cambodia, Indonesia, and Myanmar. EU as the whole accounted for the largest share in the total export values of Vietnamese footwear. The US came in second place with around 23%.

Surprisingly, in terms of export value, ASEAN is still a small market for Vietnam's footwear export when there is only around 2% of the total export value coming from this region. All in all, while the US and EU markets dominated the proportion of total export values with more than 60% in 2010, the rest is from other markets in the world.

Regarding firm's ownership type, Table 2 shows that foreign-directed investment (FDI) companies, those that either establish business operations or acquire a business asset in another country, generated the largest revenue in the market of Vietnam's footwear exports. These firms' export values accounted for approximately 65% of the total and on a rising trend. Concerning private firms, defined as firms which are owned by local individuals, their export value increased throughout years, but as a percentage to the whole, private companies' share of total export value has sunk from 33.2% in 2006 to 28.6% in 2010. Finally, state-owned enterprises (SOEs)'s export value peaked at 5.3% of the total export value in 2008, and it is in a declining trend.

**Table 2:** Export value by type of firm ownership from 2006-2010 *(Unit: million USD; Source: General Department of Vietnam's Customs).*

Types of firm's ownership	2006		2007		2008		2009		2010	
	Export value	% of total	Export value	% of total	Export value	% of total	Export value	% of total	Export value	% of total
SOEs	123	3.3	143.1	3.6	291	5.3	123	3.0	66.3	1.2
Private	1230	33.2	1125	28.8	1730	30.0	1329	32.4	1460	28.6
FDI	2303	63.5	2621	67.6	3346	64.7	2586	64.6	3616	70.2

Table 3 indicates the number of firms by types of ownership in which the number of SOEs in the footwear sector decreased from 44 in 2006 to 15 in 2010.

**Table 3:** Number of firms by types of ownership (*Source: General Department of Vietnam's Customs)*.

Types of firm's ownership	2006		2007		2008		2009		2010	
	No of firms	% of total	No of firms	% of total	No of firms	% of total	No of firms	% of total	No of firms	% of total
SOEs	44	9.4	33	7.7	26	6.9	25	6.5	15	4.1
Private	252	54.1	239	56.1	189	50.8	197	51.4	249	69.3
FDI	170	36.5	154	36.2	157	42.3	161	42.1	95	26.6

As seen in Table 3, private firms dominate the sector in terms of the number of firms, accounting around 55% of the total in 2006 and 69% in 2010. The upward trend of the number of private firms is similar to the trend in their share in export value (Table 2). On the other hand, the number of FDI firms was smaller than that of private firms. However, FDI firms enjoyed the largest share in export value.

**Fig. 4:**
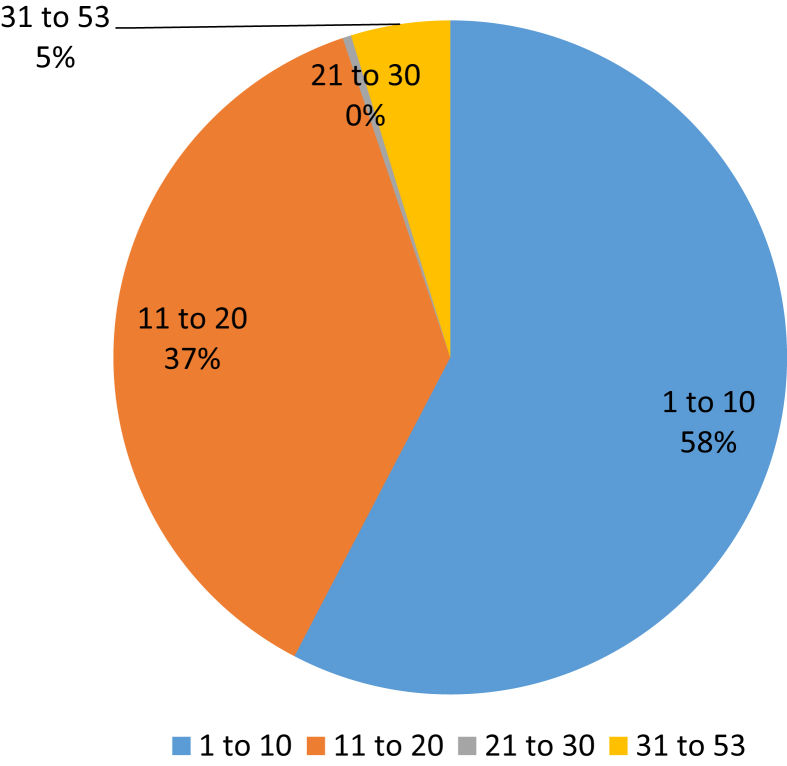
The age of footwear firms (*Unit: %; Source: Authors' calculation using Stata 14*).

The age of Vietnamese footwear firms ranged from 1 to 53 years. From Fig. 4, nearly 60% of export firms are young companies, with less than ten years of age. Firms with age from 11 to 20 years account for 37% of the total. Notably, a vast majority of firms falling in these categories are private and FDI enterprises. Older firms with more than 20 years of age accounted for only 5% and are mostly SOEs.

From Table 4, one can see that FDI firms continue to dominate regarding their export values by destination market*.* Both private and FDI firms did not focus on ASEAN market but export to very important markets such as the EU and the US. In contrast, SOEs did not concentrate on the US market obtaining the export value of only USD 2.46 million over the period while the EU and ASEAN became their major importing country groups respectively.

**Table 4:** Export values by types of ownership to destination markets from 2006-2010 (*Unit: million USD; Source: General Department of Vietnam's Customs*).

Types of firm's ownership	US	EU	ASEAN	NUEA
SOEs	2.46	72.52	12.32	61.72
Private	108.24	459.44	24.66	655.94
FDI	745.36	926.08	102.92	1089.7

## 4 Results

### 4.1 Export value, firm age, firm productivity, and number of products

Table 5 present the estimated results from applying the pooled ordinary-least-squares (OLS) approach for the multinomial logistic regression model with data over the 2006–2010 period. The value of exports in VND adjusted by GDP deflator is shown in the form of natural logarithms, and all coefficients are corrected for standard errors.

**Table 5:** Estimated results for dependent variables: US, EU, ASEAN in the 2006–2010 period.

Independent variables	US	EU	ASEAN
Exportvalue_*ijt*_	0.155*** (4.48)	0.158*** (2.7)	-0.117*** (-3.35)
Numproduct_*ijt*_	0.0467 (1.41)	-0.0716 (-1.46)	0.0975** (2.30)
Productivity_*it*_	0.227*** (8.88)	-0.0540 (-1.26)	0.187*** (3.58)
Firmage_*it*_	-0.318*** (-9.50)	-0.0516 (-0.93)	-0.115** (-2.50)
Privates	0.281*** (5.13)	-0.271*** (-4.03)	0.0613 (0.41)
FDIs	-0.341*** (-2.50)	-0.774*** (-4.95)	0.293 (1.16)
Constant	-9.297*** (-7.31)	-2.013*** (-1.34)	-3.205*** (-2.65)
Observations	21370		
Year dummies	318.55***		
Wald chi2(30)	2052.66		
P > chi2	0.000		
Pseudo R^2^	0.027		

*Notes:* t-statistics in parentheses *p < 0.10, **p < 0.05, ***p < 0.01. Pooled cross sectional multinomial logit regression with robust standard errors.

***Export value*** is positively correlated (*p* < 0.01) with the trade flows from Vietnam to the US and European countries from 2006 to 2010, while it is negative and significant at 1% for exporting to ASEAN countries. In other words, footwear firms with high export value are more likely to be attracted to the US and European markets rather than ASEAN. The US and EU are known to capture a major market segment of the international market, and it seems that Vietnam has achieved a degree of expertise in trading footwears with the US and the EU (Table 6). The results here address RQ2.

**Table 6:** Footwear export values (EV) to major markets, 2006–2010 (*Unit: million USD; Source: General Department of Vietnam's Customs*).

Country groups	2006		2007		2008		2009		2010	
	EV	% of total	EV	% of total	EV	% of total	EV	% of total	EV	% of total
US	793	22.7	866	22.2	1028	21.7	881	21.6	1244	24.3
EU	1241	35.5	1487	38.1	1718	36.6	1220	30	1624	40.0
ASEAN	61.9	1.8	125	3.2	57.4	1.2	103	2.5	70.7	1.4
NUEA	1560	40.0	1410	36.5	1930	40.5	1933	45.9	2203	34.3

**Regarding the number of products,** our analysis shows firms’ commodity diversification affects the possibility of exporting to the EU negatively. Meanwhile, it is positive but insignificant for firms serving the US market. The product diversification factor is positively correlated with exporting to the ASEAN market (*p* < 0.05). To put it differently, firms with heterogeneous products tend to choose the ASEAN market to export. Funke and Ruhwedel (2001) found that export diversification is expected to have a positive connection with the growth of transition economies. Similarly, Vietnam is experiencing product diversity when exporting to demanding markets. Here, RQ3 has been addressed, the implications will be furthered discussed in the last section.

Regarding the effects of firm age and productivity (RQ5), in line with a variety of papers implying that more productive firms could reach more distant and large markets (Bastos and Silva, 2010; Bernard and Jensen, 2004; Bernard and Wagner, 1997; Bigsten et al., 2000; Clerides et al., 1998; Fernandes and Isgut, 2005; Muûls and Pisu, 2009; Wagner, 2007), our findings indicate that the higher a firm's revenue per employee ratio, the more likely the firm exports to the US and ASEAN (*p* < 0.01). The pattern is unclear for the EU market.

A negative sign of **firm age** is found in all markets including both the distant markets such as the US and the surrounding market such as the ASEAN. While it found to be significant for the case of firms exporting to the US and ASEAN at 1% and 5% respectively, it is insignificant when it comes to the EU market. Many studies on determinants of exports show no effect of firm age concerning export performance (Iyer, 2010; Papadogonas et al., 2007; Rankin et al., 2006; Robson et al., 2012; Sousa and Bradley, 2009). There are some possible explanations for the mixed effect of firm age. On the one side, a long-established firm is more likely to accumulate managerial skills, financial capacity, and understanding of foreign markets. In contrast, young firms may not have enough experience to compete with their larger international rivals when launching a global competition campaign. On the other side, although older firms should be more efficient through their learning-by-doing process (Amornkitvikai et al., 2013), younger firms tend to be more dynamic, thus finding it easier to adapt to changes in the law and business environment overseas.

### 4.2 Types of ownership

**Ownership of firms** may influence selection their destination markets, as there are institutional and organizational differences among the FDI, privately-owned and state-owned firms. In this section, we will first present the results when all three types of ownership are included in the dataset, then we test whether our regression results remain robust if firms with different types of ownership are left out of the dataset in sequence. The test is necessary due to the high export value of the FDI footwear firms in all markets (Tables 2 and 3). As such, this section will provide a comprehensive answer for RQ4.

Generally, we find that SOEs in Vietnam get official priority, i.e., they tend to benefit from governmental financial support in the form of a lower corporate tax rate and easier access to state funds and real estate (Ngo, 2016; Vuong, 2014; Vuong and Napier, 2014). There also exists a positive but insignificant sign of trade flow from Vietnam to the ASEAN in private and FDI sectors. Notably, private ownership tends to be positively associated with exporting to the US rather than foreign-invested firms. Foreign-invested firms are more effective in improving their export performance (Aggrey et al., 2010; Farole and Winkler, 2011; Özçelik and Taymaz, 2004; Van Dijk, 2002).

Table 7 presents the results of testing the robustness of our regression results when including all three types of ownership in the dataset, and show a consistency with those mentioned in the previous section. When excluding both privately-owned and FDI firms from the sample, the statistically significant level of export value indicator reduces from 1% at all markets to 1%, 5% and 10% in the US, EU, and ASEAN respectively. It also shows in Tables 5 and 7 that SOE firms have a stronger possibility to export to the US as the coefficient of this indicator changes from 0.155 to 0.376. Remarkably, while the product diversification indicator shows a positive association for the ASEAN market, it is significantly negative if SOE firms seek to enter the EU market. The finding is in line with the literature that SOE firms seem to be less effective than the others when attempting to diversify export product items (Aggrey et al., 2010; Farole and Winkler, 2011; Javalgi et al., 2000; Özçelik and Taymaz, 2004; Rankin et al., 2006). For firms serving the US market, as the sign of product diversification changes from positive to negative with the coefficient from 0.0467 to -0.0508 and is consistently insignificant. Besides, the robustness test result shows that firm age is no longer a significant factor that hampers the probability of SOE firms to export to the ASEAN. In other words, there is not enough evidence to state that the younger firms are less likely to export to the ASEAN market and vice versa. However, one important point to note in Table 7 is that firms with low productivity serving the EU market are SOEs.

**Table 7:** Regression results with different types of firms’ ownership.

Independent variables	(1) SOEs			(2) SOEs and private firms		
	US	EU	ASEAN	US	EU	ASEAN
Exportvalue_*ijt*_	0.376*** (7.38)	0.124* (1.90)	-0.0733** (-2.48)	0.0173 (0.58)	0.182*** (3.23)	-0.194*** (-3.19)
Numproduct_*ijt*_	-0.0508 (-1.27)	-0.0851* (-1.66)	0.0489 (1.20)	0.0625 (1.15)	-0.0373 (-0.49)	0.201** (2.08)
Productivity_*it*_	0.243*** (9.12)	-0.0956** (-2.44)	0.144*** (3.01)	0.213*** (7.37)	-0.0239 (-0.49)	0.195*** (3.03)
Firmage_*it*_	-0.496*** (-12.69)	-0.0159 (-0.33)	-0.0933 (-1.62)	-0.194*** (-4.68)	-0.0614 (-0.95)	-0.113* (-1.84)
Privates	0 (.)	0 (.)	0 (.)	0.335*** (6.25)	-0.265*** (-3.73)	0.0301 (0.20)
FDIs	0 (.)	0 (.)	0 (.)	0 (.)	0 (.)	0 (.)
Constant	-14.01 (-8.08)	-1.349 (-0.88)	-2.927** (-2.56)	-6.504*** (-5.50)	-3.041** (-2.05)	-2.006 (-1.25)
Observations	11151			10219		
Year dummies	445.8***			343.55***		
Wald chi2(24)	1505.18			1211.19		
P > chi2	0.0000			0.0000		
Pseudo R^2^	0.0191			0.0304		

When only SOEs and private enterprises are included in the dataset, we do not find any change of sign in all indicators. Nevertheless, export value is now not a significant factor that affects export trade flow from Vietnam to the US while it is a significant predictor for the EU and ASEAN markets. Also, the product diversification indicator also loses its role in the EU market when the insignificant rate changes are found, meaning that it is uncertain that heterogeneous firms tend not to export to the EU. More importantly, both SOEs and private firms become influential determinants for exporting to the US at 1% significant emphasizing that both types of business ownership in the model have a high probability of exporting to the US.

## 5 Discussion and conclusion

Our findings show that there are systematic differences among the determinants of exports to the US, EU and ASEAN markets. First, the regression results show that footwear firms with high export value are more likely to target to the US and EU markets. This pattern seems quite surprising, given that all three are big markets and that the geographical distance from both the US and EU to Vietnam is bigger than that from the ASEAN to Vietnam. However, given that the ASEAN is an emerging market, perhaps, rules and regulations are not as established and easy to follow compared to those in the US and EU. Another possible hinderance for Vietnamese footwear firms to enter the ASEAN market is differences in culture and language among the ten bloc members (Rappa and Wee Hock An, 2006).

In terms of product diversification, the regression results show that the ASEAN market is positively correlated (*p-value* < 0.05), while there is no significant pattern for US and EU. As the vast cultural differences among ASEAN countries would require exporting firms to this market to diversify their products to suit the demands for each country, this finding makes intuitive sense. Together with the result concerning trade value, the extra efforts to understand the markets in different countries as well as to create products meeting the demand in each market have prevented Vietnamese footwear firms from achieving high export value in the ASEAN region, in spite of its close proximity.

Considering how the productivity of exporting firms is associated with the market choice, our findings show that a firm's revenue per employee ratio is positively correlated with exports to the US and ASEAN (*p* < 0.01).This result added evidence to the literature, which suggests that more productive firms could reach more distant and large markets (Bastos and Silva, 2010; Bernard and Jensen, 2004; Fernandes and Isgut, 2005; Muûls and Pisu, 2009; Wagner, 2007). As for firm age, our study shows this factor is negatively associated with exports to US (*p-value* < 0.01) and ASEAN (*p-value* < 0.05). This negative effect of firm age on both the distant market and the near market suggests the presence of other mitigating factors, which should be explored by other studies.

Finally, considering the impacts of the types of ownership on market choice, interestingly, we find both private ownership and FDI to be negatively correlated with export to the EU compared with their counterparts SOEs, whereas state-owned firms are more likely to export to the US. Moreover, FDI firms are negatively associated with export to both EU and the US. Private ownership is positively correlated with export to US and negatively correlated with export to EU. The results disconfirm our expectation that FDI firms would be more active in dealing with distant markets such as Europe and the US (RQ4).

In sum, the current paper analyzes various determinants of Vietnam's footwear firms' market selection. Our results imply Vietnamese footwear firms can improve their contribution to Vietnam's GDP by improving their performance in the ASEAN market. If the ASEAN community becomes more integrated as an economic zone, hence, market rules and regulations would become more easier to follow, and that would potentially compensate for the language and cultural barriers of increase export value. Here, the government of Vietnam can facilitate this integration process.

Future studies can investigate in details other psychological and cultural factors that can influence firms' market choices, as these factors are shown to be crucial in explaining economic behaviors (Vuong et al., 2018; Vuong and La, 2019). Another suggestion is to apply the Bayesian approach, which could verify the robustness of the results in this paper (La and Vuong, 2019). Last but not least, understanding the determinants of export firms’ performance can be crucial for a transitioning and globalized economy such as Vietnam, as such, it can inform the policy-making process and prevent failed implementation of international trade strategy (Vuong, 2018).

## Declarations

### Author contribution statement

Quan-Hoang Vuong: Performed the experiments; Analyzed and interpreted the data; Contributed reagents, materials, analysis tools or data; Wrote the paper.

Thi-Hanh Vu, Quang-Hung Doan: Conceived and designed the experiments; Performed the experiments; Analyzed and interpreted the data; Contributed reagents, materials, analysis tools or data.

Manh-Tung Ho: Analyzed and interpreted the data; Contributed reagents, materials, analysis tools or data; Wrote the paper.

### Funding statement

This work was supported by the Swiss State Secretariat for Economic Affairs under the SECO/WTI Academic Cooperation Project, World Trade Institute, University of Bern, Switzerland and Foreign Trade University, Vietnam under the International Trade, Trade and Investment Liberalization Project.

### Competing interest statement

The authors declare no conflict of interest.

### Additional information

No additional information is available for this paper.
